# Extended Panniculectomy as a Bridge to Renal Transplantation in a Patient With Morbid Obesity: A Case Report

**DOI:** 10.7759/cureus.100497

**Published:** 2025-12-31

**Authors:** Erick M Hernández-Mancillas, Carina M Álvarez-Dávalos, Héctor Álvarez-Trejo, Quitzia L Torres-Salazar

**Affiliations:** 1 Surgery, Universidad Autónoma de Durango, Durango, MEX; 2 Surgery, Centro Universitario de Ciencias de la Salud, Universidad de Guadalajara, Guadalajara, MEX; 3 Plastic and Reconstructive Surgery, Universidad de Guadalajara, Guadalajara, MEX; 4 Biomedical Sciences, Universidad Juárez del Estado de Durango, Durango, MEX

**Keywords:** end-stage renal disease, extended panniculectomy, kidney transplant candidate, morbid obesity, pre-transplant optimization

## Abstract

Panniculopathy associated with morbid obesity can significantly hinder access to kidney transplantation in patients with end-stage renal disease (ESRD), particularly when excessive abdominal volume and overhanging tissue compromise exposure of the iliac fossa. Extended panniculectomy has emerged as a practical pre-transplant optimization strategy, capable of improving anatomic conditions and restoring eligibility in carefully selected candidates.

We report the case of a man on hemodialysis whose severe abdominal panniculopathy precluded continuation in the transplant protocol. Despite his markedly elevated body mass index (BMI) and functional limitations, preoperative evaluation demonstrated hematologic stability and multidisciplinary clearance for major surgery. The patient underwent an extended panniculectomy, with en-bloc resection of a massive abdominal pannus and rectus muscle plication. The procedure was completed without hemodynamic instability, and the postoperative course was favorable, characterized by adequate flap perfusion, progressive reduction in drain output, early ambulation, and absence of infectious or ischemic complications. Functional improvement included enhanced mobility, improved local hygiene, and better tolerance to abdominal compression. Correction of the lower abdominal contour subsequently allowed safe surgical access to the iliac fossa, enabling reinstatement into the transplant evaluation pathway.

This case report highlights extended panniculectomy as an effective bridge procedure when abdominal morphology constitutes a modifiable barrier to kidney transplantation, rather than an absolute contraindication.

## Introduction

Severe obesity remains one of the most significant barriers to kidney transplantation, as higher body mass index (BMI) is consistently associated with increased perioperative morbidity, impaired wound healing, higher infection rates, and reduced graft survival. Most transplant programs worldwide consider obesity - particularly BMI ≥40 kg/m² - a relative or even absolute contraindication for listing, given the well-documented challenges in surgical access, postoperative recovery, and long-term graft outcomes in this population [1]. Beyond excess weight alone, many patients develop a massive abdominal pannus, especially following years of progressive obesity or incomplete weight-loss attempts. This redundant tissue constitutes not only a mechanical obstacle to the iliac fossa incision required for renal graft placement but also a high-risk zone for moisture, maceration, chronic infection, and subpannicular dermatitis, all of which markedly increase the likelihood of postoperative complications, wound dehiscence, and delayed healing [2]. Consequently, the abdominal panniculus itself often becomes an anatomical and functional contraindication for transplantation, even in patients whose metabolic comorbidities are otherwise controlled [3].

In recent years, extended panniculectomy has emerged as a pragmatic surgical strategy to optimize high-risk candidates who would otherwise remain ineligible for renal transplantation. Unlike bariatric surgery - which requires a prolonged time to achieve meaningful weight reduction - panniculectomy provides immediate anatomical benefits: it reduces the volume and weight of the pannus, improves local hygiene, lowers bacterial load, facilitates postoperative mobility, and creates a safer, more accessible operative field for future graft implantation [4]. Several international series have demonstrated that panniculectomy can safely convert previously excluded patients into eligible transplant candidates, with low rates of major complications when performed in a controlled, multidisciplinary environment [5]. Despite these advances, reports from Latin America remain scarce, and there is limited regional evidence addressing the feasibility, safety, and clinical impact of panniculectomy as a bridge procedure in patients with morbid obesity and end-stage renal disease (ESRD).

In this report, we present a case of a patient with class III obesity and severe abdominal panniculopathy who underwent extended panniculectomy as part of a pretransplant optimization strategy. This case highlights the role of body-contouring surgery in restoring transplant candidacy in anatomically complex patients within a Mexican clinical setting, contributing to the growing international experience supporting panniculectomy as a bridge to kidney transplantation. This case has been reported in accordance with the SCARE 2025 guidelines [6].

## Case presentation

**Figure 1:**
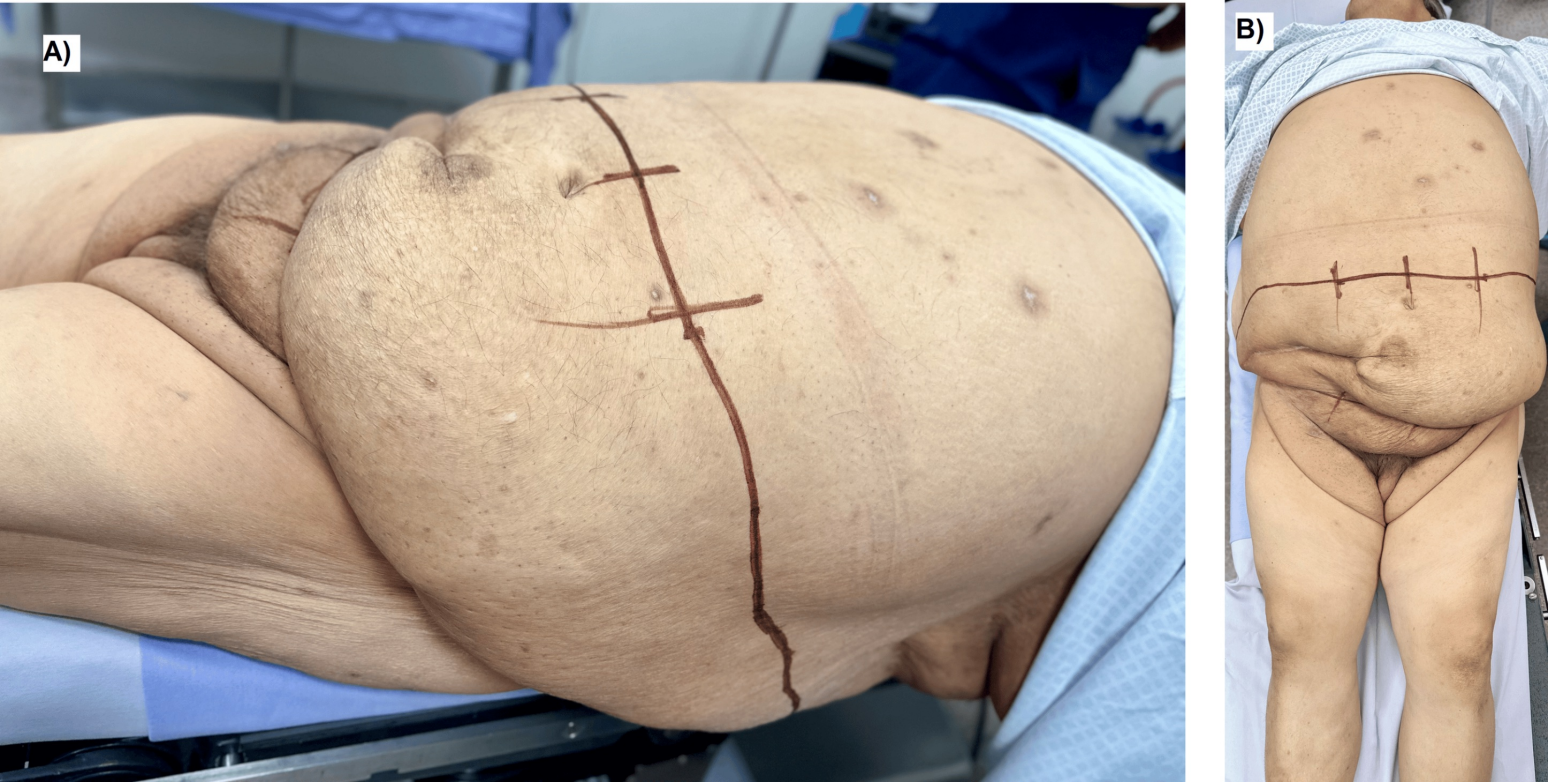
Preoperative appearance of the massive abdominal pannus in a patient with end-stage renal disease and morbid obesity. A) Lateral view demonstrating the severe ptosis, projection, and redundant overhanging tissue that obscured the lower abdominal contour and compromised potential exposure of the iliac fossa required for renal transplantation. Preoperative markings delineate the planned infraumbilical resection pattern. B) Frontal view showing the extent of lipodystrophy, skin laxity, and pannus descent, with marked distortion of the lower abdomen and groin region. These anatomic features constituted a mechanical contraindication to transplant incision placement.

A 54-year-old male, weighing 164 kg and measuring 170 cm (BMI 56.75 kg/m²), blood group O Rh-negative, was referred to the Plastic Surgery Department for evaluation of a massive abdominal pannus that impeded his eligibility for renal transplantation. His medical history included controlled systemic arterial hypertension and ESRD, managed with thrice-weekly hemodialysis via a right upper-extremity arteriovenous fistula, as well as severe abdominal lipodystrophy and symptomatic panniculopathy. No medication allergies were documented. On physical examination, the abdomen was markedly globose, with a large ptotic pannus, significant cutaneous laxity, and extensive lipodystrophy (Figures 1A, 1B).

The umbilicus appeared everted and lateralized, and a 13-cm surgical scar was observed in the right hypochondrium. Deep palpation was limited due to the thickness of the abdominal wall, and the pannus interfered with hygiene and mobility. Laboratory evaluation showed hemoglobin, hematocrit, leukocyte count, and platelet levels within normal ranges, while glucose was slightly elevated, and serum urea and creatinine were markedly increased, consistent with ESRD. Serum sodium was mildly decreased, potassium remained within normal limits, and coagulation studies - including prothrombin time (PT), international normalized ratio (INR), and activated partial thromboplastin time (aPTT) - were within acceptable parameters for major elective surgery. No preoperative imaging was reported, and no abdominal hernias were documented (Table 1).

**Table 1:** Preoperative laboratory parameters of the patient undergoing extended panniculectomy. Preoperative laboratory values of the patient, demonstrating hematologic stability to proceed with major elective surgery. Reference ranges are provided for comparison.

Laboratory parameter	Patient value	Reference range
Hemoglobin	14.1 g/dL	13.5-17.5 g/dL
Hematocrit	45.4%	41-53%
Leukocytes	9,100/µL	4,000-11,000/µL
Platelets	200,000/µL	150,000-400,000/µL
Glucose	112 mg/dL	70-110 mg/dL
Urea	58.3 mg/dL	10-50 mg/dL
Creatinine	8.3 mg/dL	0.6-1.3 mg/dL
Sodium	131 mmol/L	135-145 mmol/L
Potassium	3.9 mmol/L	3.5-5.0 mmol/L
Prothrombin time (PT)	14.5 s	11-15 s
International normalized ratio (INR)	1.08	0.8-1.2
Activated partial thromboplastin time (aPTT)	39.7 s	25-35 s

**Figure 2:**
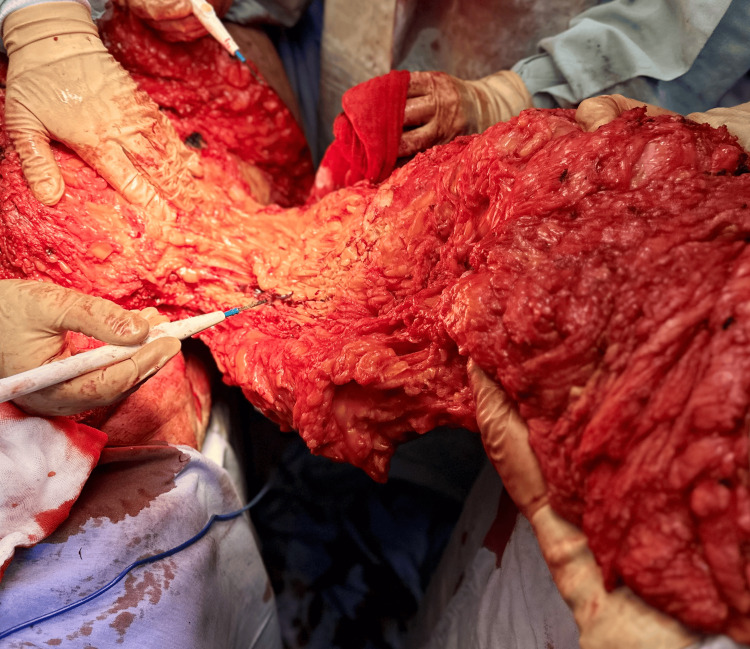
Intraoperative view showing dissection of the panniculus and delineation of the planned resection flap, highlighting the substantial subcutaneous thickness and the extent of the tissue to be removed.

**Figure 3:**
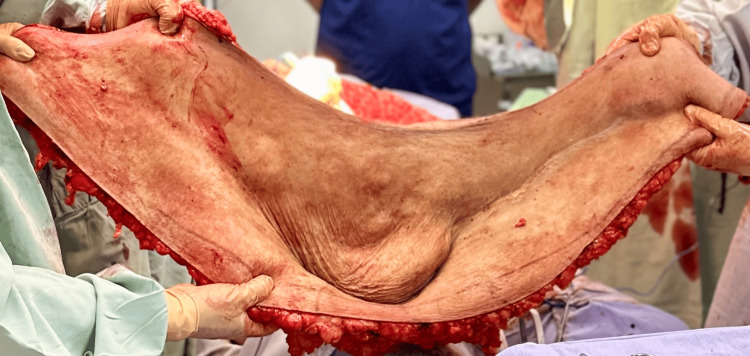
En-bloc resection of the massive abdominal pannus during extended panniculectomy. The excised specimen measured approximately 110 × 50 cm and weighed 10 kg, illustrating the extent of redundant skin and adipose tissue that created a mechanical barrier to safe iliac fossa exposure for kidney transplantation. The inferior aspect shows extensive subcutaneous adiposity and dermal thickening, consistent with chronic panniculopathy. Removal of this large pannus was essential to restore adequate abdominal contour and reduce wound-related risks before re-entry into the transplant evaluation pathway.

After a multidisciplinary assessment, the nephrology determined that the patient was clinically stable to undergo surgery as part of pretransplant optimization. An extended panniculectomy was performed under general anesthesia. Following wide antisepsis, a transverse infraumbilical incision, measuring approximately 110 × 50 cm, was created, located 4 cm above the genital border. En-bloc resection of excess skin, subcutaneous tissue, and the umbilicus yielded a 10-kg specimen (Figures 2, 3).

**Figure 4:**
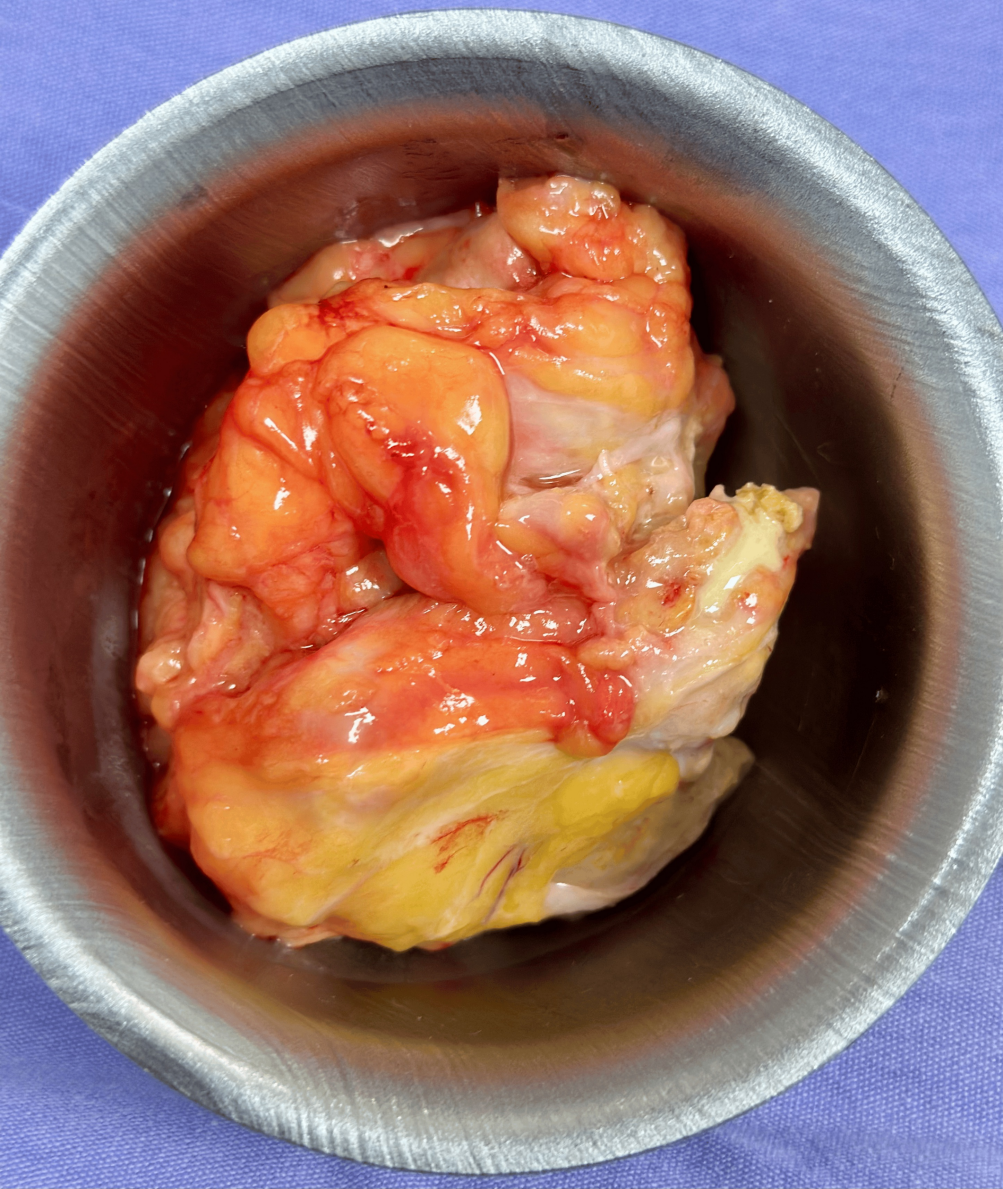
Chronic abscess cavity identified within the panniculus. Resected fibroadipose tissue containing fibrino-purulent material consistent with a chronic abscess encountered during panniculectomy.

Rectus muscle plication was performed using Prolene 1-0 in four X-shaped sutures, and two Blake drains were placed bilaterally and secured with Vicryl 1-0. Layered closure was achieved with absorbable sutures in the deep tissue and a continuous intradermal suture for skin closure. During dissection, a fibrotic cavity containing fibrino-purulent material, compatible with a chronic abscess, was identified, drained, and sent for pathological analysis (Figure 4).

**Figure 5:**
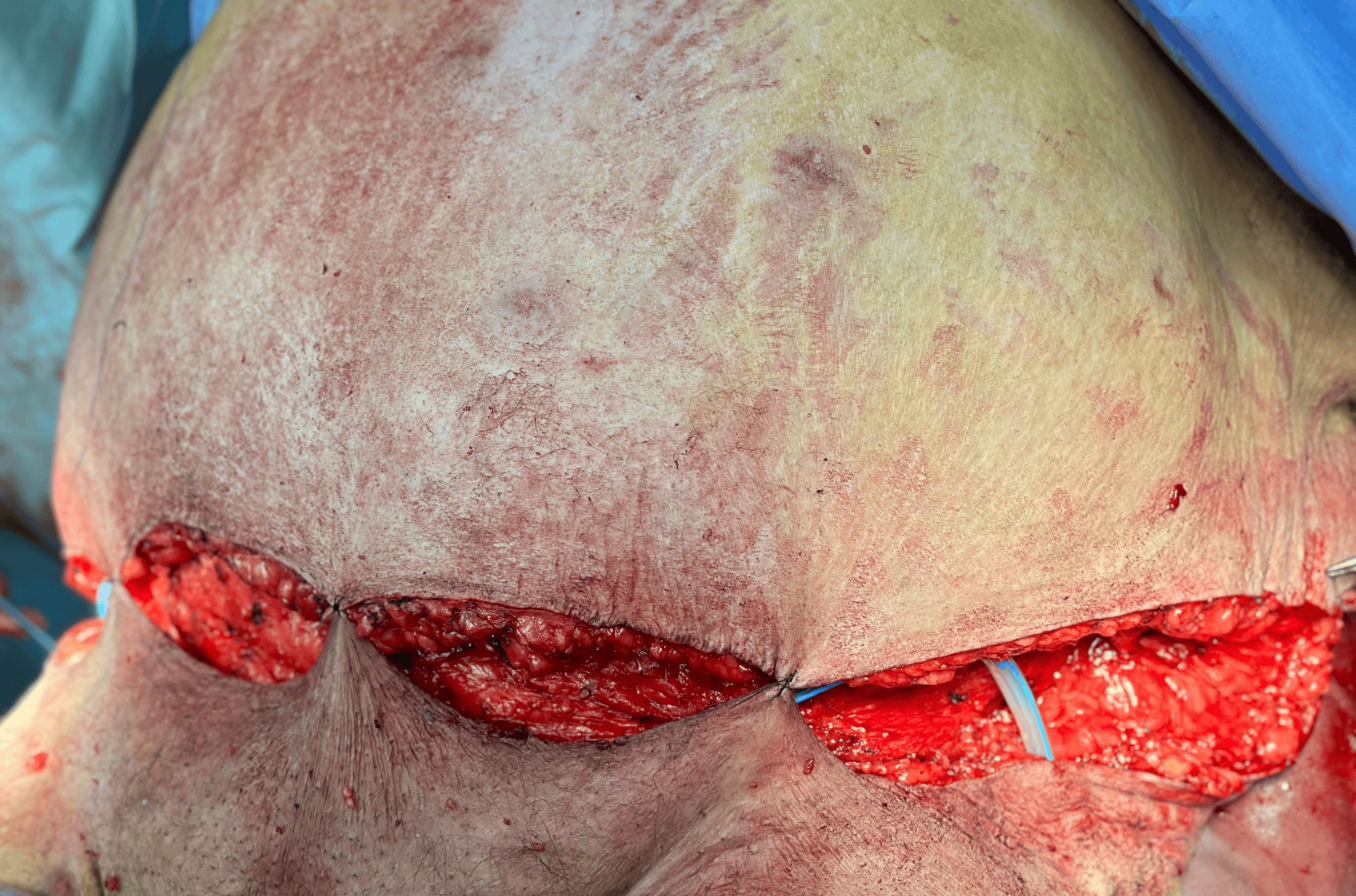
Immediate postoperative view following extended panniculectomy. Closure of the superior and inferior flaps is shown after en-bloc resection of the panniculus, illustrating flap alignment, subcutaneous tissue approximation, and placement of lateral drains prior to definitive skin closure.

Estimated intraoperative blood loss was 900 mL, with no transfusion required. The patient remained hemodynamically stable throughout the procedure. Postoperatively, combined Blake drain output in the first 24 hours measured 350 mL of serohematic fluid, decreasing progressively over subsequent days (Figure 5).

**Figure 6:**
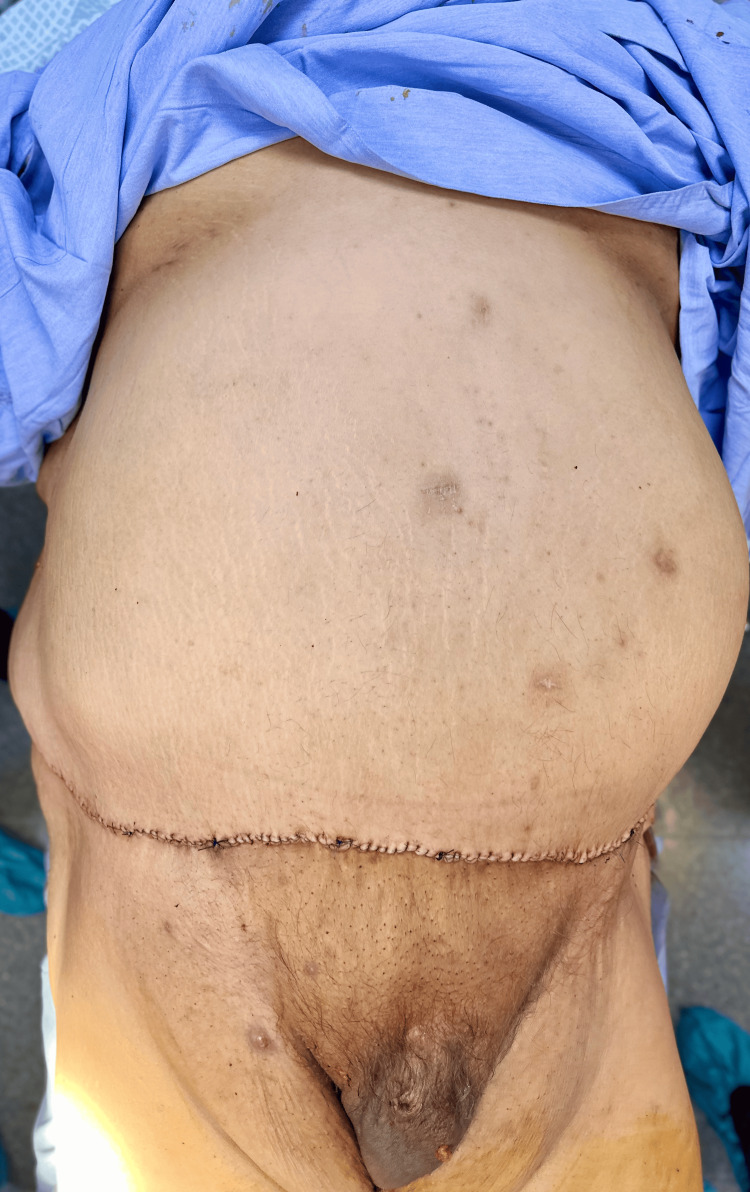
Immediate postoperative results following extended panniculectomy. Frontal view demonstrating primary closure of the abdominal flap, with good perfusion and tension-free approximation of the wound edges, creating an improved lower abdominal contour suitable for future iliac fossa surgical access.

The wound maintained adequate perfusion without signs of ischemia, necrosis, infection, hematoma, or fluid collection. Assisted mobilization began on postoperative day 2, and the patient tolerated abdominal compression adequately. Drains were removed once output decreased to less than 50 mL in 24 hours. The reduction of abdominal volume resulted in immediate improvements in mobility and local hygiene, and provided suitable anatomical conditions for future iliac fossa access (Figure 6).

Following multidisciplinary reevaluation, the patient regained eligibility for kidney transplantation. The patient expressed satisfaction with the functional improvement and the possibility of being reconsidered for transplantation. Written informed consent was obtained from the patient for the publication of this case report and the accompanying clinical images.

## Discussion

Morbid obesity and the presence of a massive abdominal pannus continue to be major limiting factors in eligibility for kidney transplantation, particularly among patients with advanced chronic kidney disease (CKD). Many transplant programs still use BMI thresholds of ≥35-40 kg/m² as exclusion criteria, due to the consistently documented increase in perioperative complications, higher risk of graft loss, and greater post-transplant mortality [7]. This restrictive approach often results in anatomically unsuitable candidates, despite their otherwise adequate metabolic or cardiovascular stability. In this context, extended panniculectomy has emerged as an effective adjunctive intervention, capable of modifying the mechanical and infectious risks imposed by redundant abdominal tissue and restoring transplant candidacy in selected patients [2]. Unlike bariatric surgery (which, although metabolically effective, requires prolonged periods to achieve clinically meaningful weight reduction), panniculectomy provides an immediate decrease in abdominal volume and offers functional benefits, such as improved mobility, enhanced hygiene, and reduced intertriginous dermatitis. These advantages are particularly relevant for patients with ESRD, who cannot postpone transplantation while awaiting long-term weight-loss interventions. Reports have demonstrated that resection of large panniculi reduces the incidence of surgical-site infections, chronic maceration, ulceration, and other pannus-related complications that interfere with dialysis management and increase postoperative morbidity [8]. Findings by Ngaage et al. indicate that, in patients with BMI >40 kg/m², panniculectomy not only reduces functional body weight but also improves objective parameters used during pretransplant evaluation, reinforcing the relevance of this procedure as an optimization strategy rather than a cosmetic intervention [9].

Our case aligns closely with evidence from Promny et al., who reported that extended panniculectomy in ESRD patients significantly reduces perioperative wound complications and enables reinstatement into transplant programs, with only minor issues, easily manageable with local care [2]. Likewise, Kuo et al. demonstrated that pretransplant panniculectomy improves surgical access and decreases wound morbidity in high-risk renal transplant candidates, supporting its safety profile even in medically complex populations [8]. In our patient, the procedure resulted in anatomical correction of a high-risk panniculus, reduction of BMI-related impediments, and identification and drainage of a chronic abscess that would have posed an important risk in the context of future immunosuppression. The postoperative evolution was favorable, with adequate flap perfusion, progressive reduction in drainage, no signs of necrosis or infection, and prompt recovery of mobility - all consistent with contemporary literature describing acceptable morbidity in ESRD patients when surgery is performed under standardized technique and multidisciplinary oversight.

Another important consideration is the impact of obesity on post-transplant outcomes. Excess abdominal adiposity has been associated with a higher risk of wound dehiscence, lymphorrhea, seroma, and delayed graft exposure during the retroperitoneal or transabdominal incision used in kidney transplantation. By reducing soft-tissue volume and improving cutaneous perfusion, panniculectomy not only facilitates eligibility but may also mitigate postoperative risk, contributing to improved early surgical outcomes. Both Promny et al. and Kuo et al. emphasize that performing panniculectomy as a staged procedure (prior to transplantation) allows for wound stabilization before the initiation of immunosuppression, reducing the likelihood of complex wound complications that could jeopardize graft integrity [2,8].

Selection of appropriate candidates remains essential. Still, multiple series have demonstrated that even patients with stage 4-5 CKD can undergo panniculectomy with an acceptable perioperative risk profile when comorbidities are optimized and surgical principles, such as tension-free closure, meticulous hemostasis, and effective drainage, are followed [10]. Our case reinforces this observation, given the resection of a 10-kg panniculus (substantially larger than typical volumes reported in the literature) without major complications.

A multidisciplinary approach involving plastic surgery, nephrology, transplant surgery, and nutrition is indispensable to individualizing treatment plans and determining which patients will benefit most from pretransplant body-contouring procedures [11]. Although panniculectomy is not a substitute for long-term metabolic control, it represents a practical and immediate bridge strategy when bariatric surgery is contraindicated, unavailable, or too slow relative to the patient’s clinical urgency [9]. The present case contributes to the limited Latin American literature by illustrating the safety and feasibility of extended panniculectomy in a high-BMI ESRD patient and underscores its value as a reconstructive intervention capable of restoring transplant eligibility in anatomically complex candidates.

Extended panniculectomy represents a safe and effective surgical strategy for optimizing renal transplant candidates with morbid obesity and a massive abdominal pannus, particularly when the redundant tissue constitutes a direct anatomical contraindication to transplantation [4]. In this case, resection of a 10-kg panniculus corrected the mechanical barrier that prevented access to the iliac fossa, resolved a chronic infectious focus, and improved mobility and local hygiene, without generating major perioperative morbidity. These outcomes support the growing evidence that panniculectomy, when performed under standardized technique and multidisciplinary evaluation, can restore transplant eligibility in carefully selected patients with ESRD who are otherwise denied the opportunity for a life-saving intervention. This case report reinforces the role of panniculectomy as an effective bridge procedure, rather than a weight-loss strategy, emphasizing the importance of individualized assessment and coordinated care to expand access to kidney transplantation in anatomically complex candidates. Nevertheless, as a single-patient report, the absence of standardized pannus grading scales, objective functional metrics, and long-term follow-up represents an inherent limitation. Future prospective series and multicenter studies should incorporate validated classification systems, quantified functional outcomes, and extended follow-up to transplant listing and post-transplant wound evolution, to further define the role of panniculectomy in this high-risk population.

## Conclusions

Extended panniculectomy represents a safe and effective surgical strategy for optimizing renal transplant candidates with morbid obesity and a massive abdominal pannus, particularly when the redundant tissue constitutes a direct anatomical contraindication to transplantation. In this case, resection of a 10-kg panniculus corrected the mechanical barrier that prevented access to the iliac fossa, resolved a chronic infectious focus, and improved mobility and local hygiene, without generating major perioperative morbidity.

These outcomes support the growing evidence that panniculectomy, when performed under standardized technique and multidisciplinary evaluation, can restore transplant eligibility in carefully selected patients with ESRD who are otherwise denied the opportunity for a life-saving intervention. This case report reinforces the role of panniculectomy as an effective bridge procedure, rather than a weight-loss strategy, emphasizing the importance of individualized assessment and coordinated care to expand access to kidney transplantation in anatomically complex candidates.
